# Does improving basic public health services promote household consumption of rural migrant workers? Evidence from China

**DOI:** 10.3389/fpubh.2023.1308297

**Published:** 2024-01-08

**Authors:** Lan Pan, Gang Li, Haoran Wan

**Affiliations:** ^1^School of Economics, Huazhong University of Science and Technology, Wuhan, China; ^2^School of Economics and Management, Huazhong Agricultural University, Wuhan, China

**Keywords:** basic public health services, rural migrant workers, household consumption, health literacy, citizenization willingness

## Abstract

**Background:**

Transforming rural migrant workers’ consumption potential into a consumption booster requires ensuring their equal rights as urban residents. The adequate access to Basic Public Health Services (BPHS) help effectively tackle rural migrant workers’ health challenges and promote the well-being of this vulnerable population. Assessing the welfare effects of BPHS through a consumption perspective offers valuable insights and provides policy implications for enhancing the equity of BPHS and achieving common prosperity.

**Methods:**

Utilizing the household-level data from China Migrants Dynamic Survey 2017 (CMDS 2017), this study comprehensively evaluated the effects of BPHS on rural migrant workers’ household consumption by combining the methods of OLS, PSM, and IV.

**Results:**

The enhancement of BPHS promotes rural migrant workers’ household consumption even after considering endogeneity problems. Mechanism analysis indicates that BPHS imposes its positive effects on rural migrant workers through improving health literacy and increasing citizenization willingness. Furthermore, we identified heterogeneous effects across individual and household characteristics of rural migrant workers, and their flow patterns.

**Conclusion:**

Our analysis indicates that BPHS plays a greater role in promoting household consumption of socially vulnerable groups, such as trans-provincial migration, rural migrant workers in old generations, and with lower-level income. Overall, these results suggest that the welfare effects of BPHS are inclusive in China.

## 1 Introduction

Along with the increased uncertainty about global economic, leveraging consumption to drive economic growth is a universal challenge (1). The growing consumer demand and power of rural migrant workers have begun to change the commercial landscape of Chinese cities and promote economic transformation (2). China Migrant Workers Monitoring Survey Report in 2022 showed that there were nearly 300 million rural migrant workers in China. Therefore, transforming rural migrant workers’ consumption potential into a consumption booster is crucial in promoting economic and social development and achieving common prosperity.

Compared with the rapid growth of household income of rural migrant workers, there is still a certain gap between household consumption expenditure and consumption rate of rural migrant workers and urban residents (3). In view of this phenomenon, scholars have explored other influencing factors of consumption from multiple dimensions, including human capital (4), migration patterns (5), family structure (6), and household registration system (7). Meanwhile, it cannot be ignored that because of social, economic, and cultural marginalization, the limited access to healthcare services in inflow cities poses a threat to rural migrant workers’ welfare and then inhibits their household consumption (8, 9). Therefore, ensuring their equal rights as urban residents is the key to promoting consumption.

With the continuous improvement of Basic Public Health Services (BPHS), this vulnerable population is paid much attention to acquire sufficient services to address health challenges, promote health equity, and improve their well-being (10, 11). As a key factor in the utility function, consumption provides a unique perspective to explore the welfare effects of BPHS (12, 13). Regrettably, existing researches primarily focus on specific parts of BPHS (14–16), relatively ignore the comprehensive assessment of BPHS. According to the precautionary saving theory, rural migrant workers tend to save more due to the low level of social security (17). By providing medical insurance, future uncertainties can be better managed, which may increase current consumption (14, 18, 19). Besides, the popularization of medical insurance has greatly improved people’s health status and alleviated health disparities (9, 20, 21). The social security role of medical insurance has led to an increasing flow of peasant household into the cities. Under the improvement of consumption environment and the influence of urban consumption culture, rural migrant workers unconsciously learn and imitate the consumption habits and modes of urban residents, and increases the diversity of household consumption (22).

In this study, policy advocacy, health file, public health education, health services accessibility, and medical insurance are considered, which is conducive to a comprehensive understanding of the effects of BPHS. It is noteworthy that some scholars begin to discuss the regional and urban–rural disparities of BPHS, but still with little analysis of equality among different groups, such as rural migrant workers (23, 24). With the enhancement of BPHS, can the welfare gap between rural migrant workers and other groups be narrowed? If there is an impact, how does it work? What heterogeneity exists in different mobility patterns, generations, and income levels? This study aims to address these questions. Existing literature provides the basis for this study, but there are still the following innovations: Firstly, to the best of our knowledge, this study is among the first to systematically evaluate the welfare effects of BPHS from the perspective of household consumption of rural migrant workers, providing valuable insights for policymakers. Secondly, this study benefits from the abundant information available in the China Migrants Dynamic Survey (CMDS) dataset, enabling us to explore the possible mechanisms underlying the effects of BPHS. We find that health literacy and citizenization willingness are the main channels, offering new micro evidence from China. Finally, the heterogeneity analysis verifies the inclusive function of BPHS, which supplements the field of the welfare of socially vulnerable groups, and provides targeted approaches to promote household consumption of rural migrant workers and achieve common prosperity.

The rest of our study is structured as follows. In Section 2, we introduce the institutional background of BPHS. Section 3 outlines the theoretical framework as well as the hypotheses to be tested. Section 4 describes the data and main variables considered. Section 5 introduces the empirical strategies employed in the research. Section 6 presents and discusses the empirical results. Section 7 is the discussions and is followed by the conclusions in Section 8.

## 2 Institutional background

China has attached great importance to basic public services, especially in the field of BPHS. The development processes of BPHS can be roughly divided into three periods, including initial period, exploration and development period, and stabilization and standardization period. Table 1 summarizes the critical policy implementations of each period, and the detailed information can be seen in Supplementary material S1.

**Table 1:** Development processes of BPHS.

Period	Policy implementation
Initial period	Initiate China’s primary health service system; Launch market-oriented reforms on medical and health service system and a series of insurance schemes, such as the Urban Employee Basic Medical Insurance (UEBMI), the New Rural Cooperative Medical Scheme (NRCMS), and the Urban Resident Basic Medical Insurance (URBMI)
Exploration and development period	Issue the National Basic Public Health Service Program (NBPHS); Issue the document “12th Five-Year Plan for the Development of health Services,” which aims to ensure that by 2015 all residents would have access to basic medical security and BPHS
Stabilization and standardization period	Carry out “Equalization Program of Basic Public Health and Family Planning Services for Migrants (EHFPSM)”; Improve the system of basic public services, improve the level of public services, make them more balanced and accessible, and steadily promote common prosperity in the report to the 20th National Congress of the Communist Party of China

Based on the items of BPHS provided by Chinese government, this study mainly reflects the current situation of China’s BPHS from policy advocacy, health file, public health education, health services accessibility, and medical insurance.

### 2.1 Policy advocacy

Policy advocacy about BPHS is a key way for people to understand and use their basic rights. According to the questions in the CMDS, “Have you ever heard of the NBPHS?,” only 59.60% of rural migrant workers answered “yes” in the sample. This indicates that the publicity of inclusive policies is not strong enough, and there may exist problems such as incomplete coverage of groups and lack of pertinence.

### 2.2 Health file

**Figure 1:**
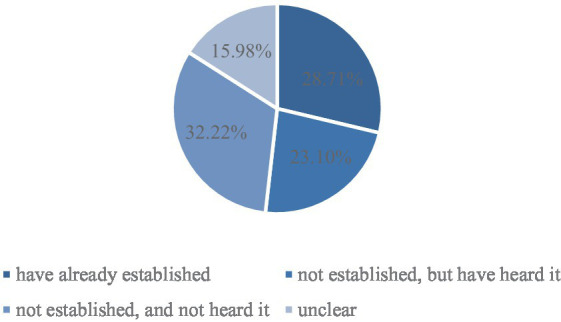
Health file establishment.

Residents’ health file is a major exploration and application of medical informatization construction, which is based on the physical and mental health of permanent residents in the area under the jurisdiction. Besides, it is an information resource that can realize multi-channel dynamic collection of information, and meet the needs of residents themselves and health management. As shown in Figure 1, in the sample, only 28.71% of rural migrant workers have established health files in inflow cities, 23.10% of them just have heard of this service, and 48.20% of them did not even know about health file establishment.

### 2.3 Public health education

**Figure 2:**
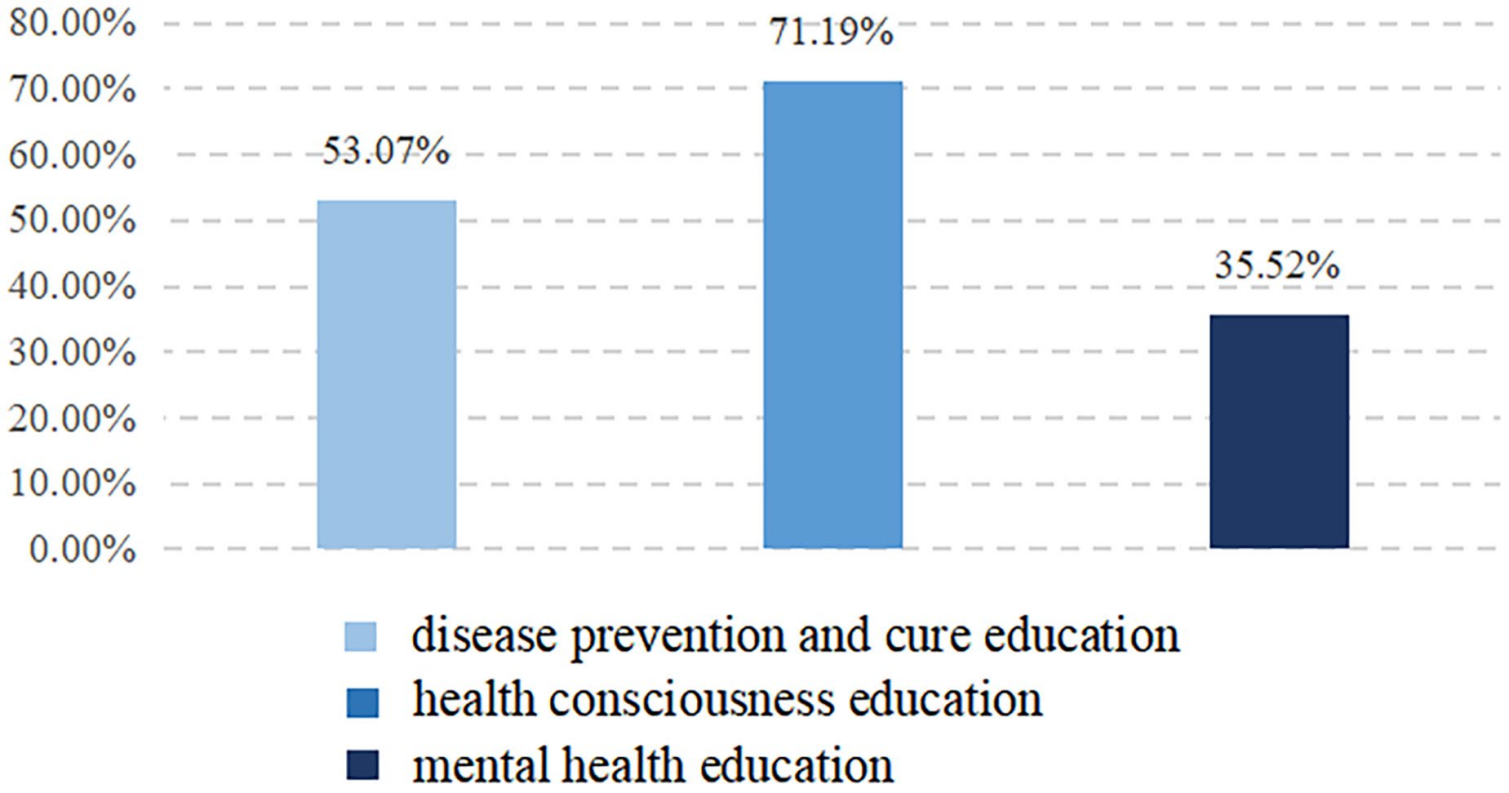
Different kinds of public health education acceptance.

Public health education enables people to consciously adopt healthy behaviors and lifestyles, eliminate risk factors, prevent diseases, and then improve health level. In the CMDS, health education includes disease prevention and cure education,^1^ health consciousness education,^2^ and mental health education. As presented in Figure 2, health consciousness education was the most popular among rural migrant workers, covered more than 70%. The second was disease prevention and cure education. Only 35.52% of rural migrant workers have received mental health education, indicating that mental health has not aroused their own and the society’s extensive attention.

### 2.4 Health services accessibility

According to the National Health Commission, the three-tier network of medical and health services covering both urban and rural areas has been improved, and 90% of families were able to reach the nearest medical point within 15 min in China.^3^ While, in this study, we found that 84.79% of rural migrant workers got to the nearest medical institution within 15 min.

### 2.5 Medical insurance

**Figure 3:**
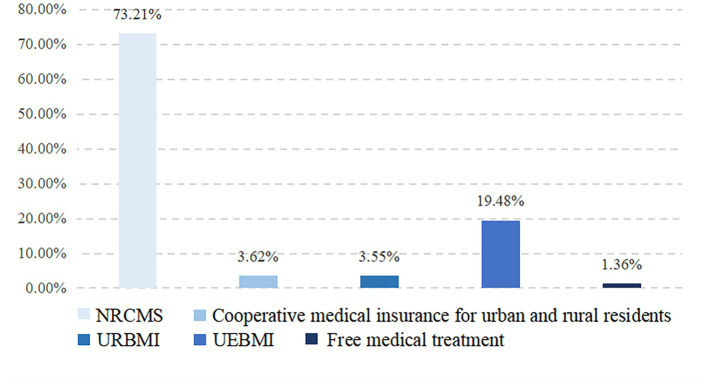
Different kinds of medical insurance participation.

Medical insurance is an important part of equalizing BPHS, and is a key to alleviating the problem of “difficult and expensive medical care.” According to the National Healthcare Security Administration, by the end of 2021, the number of people covered by basic medical insurance have reached 1,364.24 million, with coverage stable at over 95 percent.^4^ As shown in Figure 3, rural migrant workers are plagued with low participation in medical insurance targeted for urban residents.

## 3 Theoretical framework and hypothesis

### 3.1 The impact of BPHS on household consumption of rural migrant workers

The primary objective of BPHS is to protect health rights. However, due to the hukou system in China, rural migrant workers often have limited access to BPHS, which undoubtedly harms their welfare (25, 26). Fortunately, with the implementation and improvement of BPHS, government has begun to focus extensively on improving the economic and social welfare of this vulnerable population. Fiscal expenditure and funds allocation of BPHS play a crucial role in determining the degree and distribution of social security and social welfare. The high level and quality of BPHS received by rural migrant workers can provide a sense of future security, which may lead to changes in the allocation of household resources. This can result in increased consumption and decreased precautionary saving in the present (14, 26, 27). In addition, enhancing BPHS can help rural migrant workers achieve fair treatment, which can stimulate their sense of identity and belonging to the city (28). This process can also narrow the gap of lifestyles and life concepts between rural migrant workers and urban residents, thus impacting their consumption patterns and behaviors. Therefore, we propose the following research hypothesis:

*Hypothesis 1*: The enhancement of BPHS can promote rural migrant workers’ household consumption.

### 3.2 The mechanisms of BPHS on household consumption of rural migrant workers

**Figure 4:**
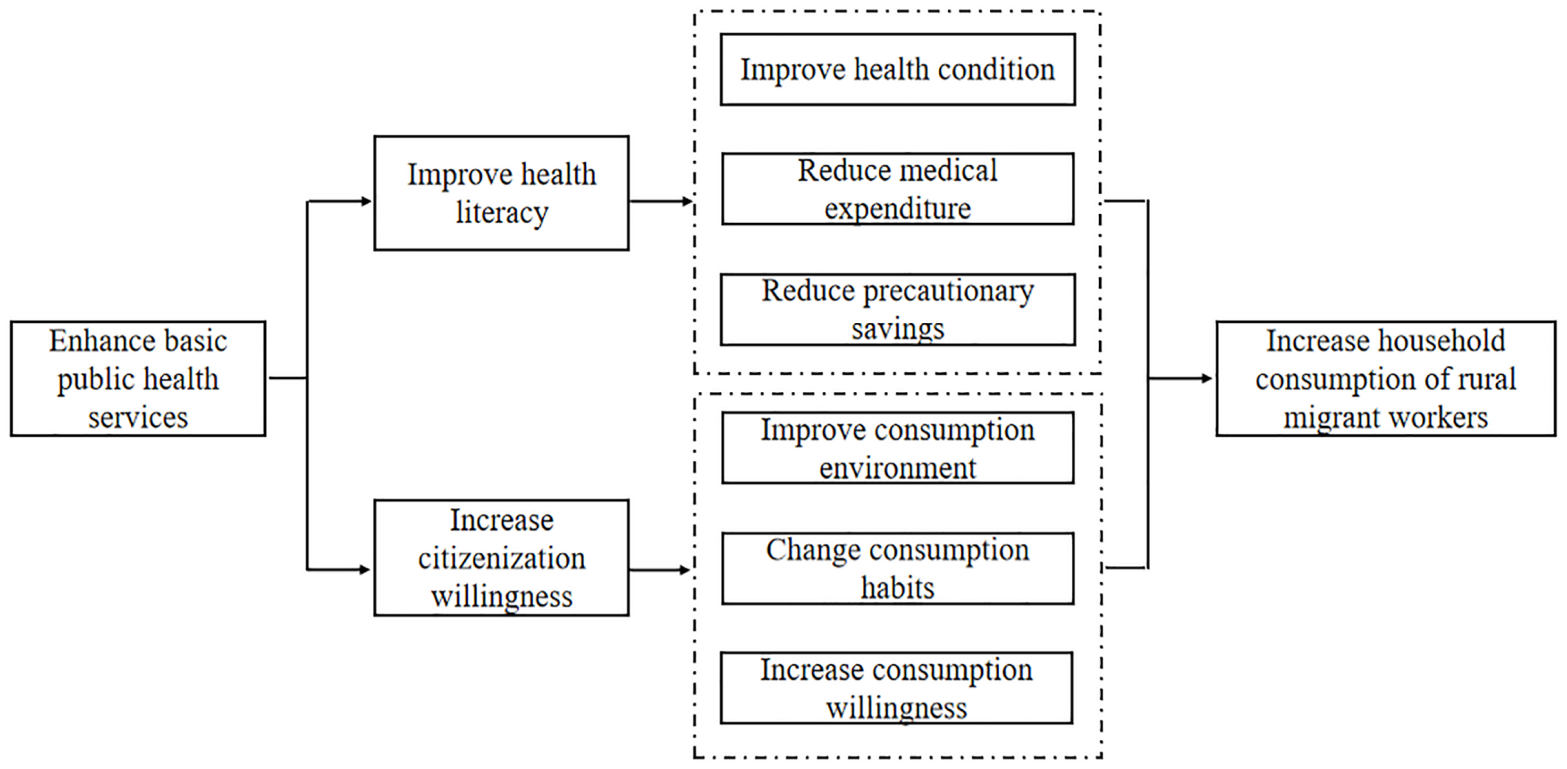
The conceptual framework.

As illustrated in Figure 4, we have classified the possible mechanisms through which BPHS affect rural migrant workers’ household consumption into the following two categories: improving health literacy, and increasing citizenization willingness.

#### 3.2.1 Improving health literacy

Health literacy is one of the important factors affecting people’s health and well-being, encompassing the ability to obtain, understand, and utilize health information and services to make behaviors and decisions that benefit their own health. As shown in Figure 4, sufficient and equal access to BPHS can enhance health literacy of rural migrant workers, leading to better health outcomes, reduced medical expenses and precautionary savings (29). Firstly, by providing health education and pamphlets, BPHS encourage timely medical treatment and ultimately reducing potential health risks. As a kind of human capital, good health condition ensures work efficiency and improves wage income, thus increasing household consumption (30, 31). Secondly, free physical examinations and medical insurance can alleviate the economic burden of medical expenses and increase the willingness to seek medical attention, resulting in more efficient utilization of medical resources, controlled health risks, and reduced crowding-out effect of medical spending on household consumption (15, 32). Finally, good health literacy can mitigate health anxiety and uncertainty, stabilize future income expectations, and reduce precautionary household savings and consumption volatility (14, 27, 33). Therefore, we propose the following research hypothesis:

*Hypothesis 2*: The enhancement of BPHS can promote rural migrant workers’ household consumption by improving health literacy.

#### 3.2.2 Increasing citizenization willingness

Based on the push-pull theory, high quality public services act as positive factors that attract population inflow (34). As shown in Figure 4, BPHS are effective forces to attract talents. When rural migrant workers feel cared in inflow cities, the psychological and social distance will be shortened, and the sense of belonging and identity will be enhanced, thus increasing their willingness to become citizens. As for the definition of citizenization willingness, scholars commonly use long-term residence intention and hukou intention to measure it. Besides, some scholars further introduce psychological identity (28). Therefore, to some extent, the citizenization willingness in this paper is significantly related to citizenization behavior. The increase of citizenization willingness undoubtedly boosts household consumption. Firstly, as consumption environment is one of the key factors for consumption decision, cities with diverse consumption scenarios for goods enhance consumption expenditure and quality compared to rural areas (22, 35). Secondly, in the process of citizenization, the traditional consumption concept, consumption consciousness, and cultural psychology of rural migrant workers are imperceptibly impacted by urban lifestyle, they may unconsciously learn and imitate the consumption habits and modes of urban residents. Finally, household registration restrictions can be effectively solved after citizenization (36, 37). In other words, rural migrant workers can receive equal protection in employment, pension, medical care, education, housing and other aspects. The improvement of consumption power and the change of consumption willingness inevitably increase the household consumption demand and their marginal propensity to consume, especially in aspects such as culture, entertainment, transportation and communication (38). Therefore, this study proposes the following research hypothesis:

*Hypothesis 3*: The enhancement of BPHS can promote rural migrant workers’ household consumption by increasing citizenization willingness.

## 4 Methods

### 4.1 Data

The data used in this study is derived from the China Migrant Dynamics Survey (CMDS) conducted by the National Health Commission. The survey focused on inflow population over the age of 15, who had resided in the inflow area for more than a month and had not registered in the district (county, city). It aimed to gather information on the demographic and economic characteristics of floating population, their range of movement, willingness to become citizens, and access to BPHS. Although CMDS data has been available for multiple periods since 2009, this study primarily used the CMDS 2017 sample due to the availability of data and the presence of unique variables, such as Policyadvocacy and Psychologicalidentity. Additionally, this data selection is consistent with some existing studies (37).

Since this study focuses on rural migrant workers, and only respondents who have lived in inflow cities for more than 6 months are required to answer questions about BPHS, the samples selected in this study should meet the following criteria: (1) have lived in inflow city for more than half a year with agricultural or transfer-from agricultural hukou; (2) age between 16 and 65;^5^ (3) migrate only for work or business and exclude unemployed. In addition, continuous variables such as consumption and income have been winsorized by eliminating samples among the top 1% and the bottom 1%, and 93,376 households are finally retained as the research objects.

### 4.2 Variables

#### 4.2.1 Household consumption

Household consumption of rural migrant workers includes all kinds of expenditure in production and life, such as food expenditure, medical expenditure, and housing expenditure. In this study, we took the logarithmic of households’ total annual consumption expenditure (Consumption) as the main dependent variable. The converted amount of food and housing covered by employment units were also included. The consumption excluding the above conversion costs (Consumption_exclude) was used for the robustness test. We also collected three items as alternative measures for household consumption of rural migrant workers, includingConsumptionrate, Averagepersonconsumption, and Averagelaborconsumption. Considering that high housing prices may affect household consumption decisions, we further tested the relationship between BPHS and Consumptionwithouthousing.

#### 4.2.2 Basic public health services

We conducted an indicator system to measure BPHS. Table 2 showed the specific indicators and their definition. The variable BPHSindex was calculated as follows. Firstly, we defined $x_{ij}$ to be the actual benefit level of rural migrant worker i in the index j. In other words, the index measured whether rural migrant workers enjoyed the same level of BPHS as urban residents. Therefore, we used 0–1 dummy variables for representation. Secondly, we adopted the entropy method to determine the weight of each indicator (39). $w_{j}$ represented the weight of index j. Finally, we calculated BPHS based on the formula $\mathrm{BPHS}\;\mathrm{inde}x_{i}=\overset{7}{\underset{j=1}{\sum }}w_{j}x_{ij}$.

**Table 2:** Indicator system for BPHS.

Indicator	Definition	Weight
Policy advocacy	1 if heard the NBPHS, 0 if have not heard the NBPHS	0.096
Health file	1 if established a local health file, 0 if have not established health file	0.232
Disease prevention and cure education	1 if received disease prevention and cure education, 0 if have not received this education	0.118
Health consciousness education	1 if received health consciousness education, 0 if have not received this education	0.063
Mental health education^a^	1 if received mental health education, 0 if have not received this education	0.192
Health services accessibility	1 if got to the nearest medical station within 15 min, 0 if got to the nearest medical station more than 15 min	0.031
Medical insurance	1 if participated in urban residents’ medical insurance, urban workers’ medical insurance or socialized medical insurance, 0 if have not participated the above medical insurance	0.269

a This study also tried to set the definition of public health education variable as “1 if received at least one public health education, 0 otherwise.” According to this standard, the proportion of migrant workers who have received public health education in the sample is as high as 73.60%. So, using such binary variable was difficult to accurately reflect the actual differences of migrant workers receiving various kinds of health education.

A-F double critical value method is always regarded as a multidimensional poverty analysis tool (40). This study reevaluated BPHS according to this method for robustness test. Referring to existing studies (4), and considering the median value and mean value of BPHSindex (Table 3), we set the critical value of access to BPHS as 0.30, that is, when BPHSindex was larger or equal to 0.30, rural migrant workers were judged to be in the multidimensional access to BPHS, and the variable BPHS_equalequaled one. In other words, rural migrant workers basically enjoyed the same rights as city dwellers. Besides, we changed the critical value of access to BPHS to 0.40 for further test in order to avoid the difference of results caused by the subjective selection of critical values.

**Table 3:** Data definition and descriptive statistics.

Variables	Definition	Mean	Std. Dev.	Min	Max
**Panel A: Household consumption (*N* = 93,376)**					
Consumption	Logarithmic of households’ total annual consumption expenditure	10.548	0.536	8.700	12.725
Consumption_exclude	Logarithmic of households’ total annual consumption expenditure without the conversion cost of accommodation and lodging	10.481	0.564	8.700	12.032
Consumption rate	The ratio of household consumption to household income	0.588	0.273	0.017	6.667^a^
Average person consumption	Logarithmic of average person consumption	9.475	0.593	6.753	12.165
Average labor consumption	Logarithmic of average labor consumption	9.769	0.590	7.090	12.165
Consumption without housing	Logarithmic of households’ total annual consumption expenditure without housing expenditure	10.241	0.841	0.000	12.700
**Panel B: BPHS (*N* = 93,376)**					
BPHS index	Based on the calculation above	0.388	0.272	0.000	1.000
**Panel C: Respondent characteristics (*N* = 93,376)**					
Gender	1 if men, 0 if women	0.590	0.492	0.000	1.000
Age	Actual age of respondent	36.620	9.455	16.000	65.000
Education	Education level of respondent: 1 if never attended school, 2 if primary school, 3 if junior high school, 4 if high school/technical secondary school, 5 if junior college, 6 if undergraduate, and 7 if postgraduate	3.283	1.020	1.000	7.000
Not married (reference group)	1 if not married, 0 if married, divorced or widowed	0.138	0.345	0.000	1.000
Married	1 if married, 0 if not married, divorced or widowed	0.841	0.366	0.000	1.000
Divorced or widowed	1 if divorced or widowed, 0 if not married or married	0.021	0.144	0.000	1.000
Party member	1 if member of the Communist Party of China, 0 if not the member of the Communist Party of China	0.036	0.186	0.000	1.000
Employed (reference group)	1 if employed, 0 if employer or self-employed	0.549	0.498	0.000	1.000
Employer	1 if employer, 0 if employed or self-employed	0.055	0.228	0.000	1.000
Self-employed	1 if self-employed, 0 if employed or employer	0.380	0.486	0.000	1.000
**Panel D: Household characteristics (*N* = 93,376)**					
Family size	Number of respondents’ family members	3.192	1.176	1.000	10.000
Local size	Number of households living with respondent in the country of departure	2.618	1.199	1.000	10.000
Old share	The proportion of older adult people in household	0.005	0.038	0.000	0.667
Child share	The proportion of children in household	0.222	0.201	0.000	0.833
Income	Logarithmic of annual household income	11.174	0.513	9.798	12.794

a The consumption rate exceeds 1 is realistic. On the one hand, if there is a loss of household production and operation, the household income will decline significantly. On the other hand, medical expenses, housing expenses and other large expenditures often make it difficult for low-income families to cover all of them with their current income. Besides, the phenomenon of premature consumption and excessive consumption is also relatively common among the new generation of rural migrant workers.

Std. Dev. refers to standard deviation.

#### 4.2.3 Control variables

Based on the existing literature and field experience, we further included three levels of control variables (5, 13, 41). First, in terms of respondent characteristics, basic information and social attitude were included, such as Gender, Age, Education, marital status (Notmarried, Married, Divorcedorwidowed), Partymember, and working status (Employed, Employer, Self−employed). Second, at the household level, economic and demographic characteristics were taken into consideration. Specifically, including Familysize, number of households living with the respondent in the country of departure (Localsize), population burden ratio (Oldshare,Childshare), and the logarithmic of annual household income (Income). Third, at the macro level, we mainly considered city fixed effects.

## 5 Models

### 5.1 Baseline model

In order to evaluate the impact of BPHS on the household consumption of rural migrant workers, this study took consumption as the dependent variable and BPHS index as the independent variable to construct a baseline model as follows:


(1) $$\mathrm{Consumptio}n_{i}=\alpha_{1}+\beta_{1}\mathrm{BPHS}\;\mathrm{inde}x_{i}+\gamma_{1}\mathrm{Controls}+\theta_{p}+\varepsilon_{1i}$$

In Equation (1), $\mathrm{Consumptio}n_{i}$ denotes the consumption of rural migrant worker’s household i. $\mathrm{BPHS}\;\mathrm{inde}x_{i}$ refers to the acquisition share of BPHS. Controls is a set of control variables, mainly including characteristics at the respondent, and household levels. $\theta_{p}$ is the city fixed effects. $\alpha_{1},\beta_{1},\gamma_{1}$ are parameters to be estimated, and $\varepsilon_{1i}$ is a random disturbance item.

### 5.2 Propensity score matching method

In order to alleviate the bias of the estimation results, this study used the Propensity Score Matching (PSM) method. According to the counterfactual analysis framework proposed by Rosenbaum and Rubin, we defined the average treatment effect on the treated group (ATT) as follows (42):

(2) $$ATT=E\left(y_{1i}|D_{i}=1\right)-E\left(y_{0i}|D_{i}=1\right)$$

In Equation (2), $D_{i}$ is a treatment variable that represents rural migrant workers’ decision. $y_{1i}$ denotes household consumption of rural migrant worker i after obtaining BPHS, while $y_{0i}$ denotes household consumption of rural migrant worker iwithout motivation to acquire BPHS. $E\left(y_{1i}|D_{i}=1\right)$ is the actual result that can be directly observed, while $E\left(y_{0i}|D_{i}=1\right)$ is the counterfactual result.

The basic logic of PSM method is to predict the probability of rural migrant workers’ access to BPHS based on observable variables, then employs neighbor matching, caliper matching and other methods to carry out propensity score matching, and finally uses the matched samples to measure the average processing effect of BPHS on rural migrant workers’ household consumption (ATT). If the estimation results of different matching methods are similar, this verifies that the results are robust.

### 5.3 Instrument variable method

There may be endogeneity problems caused by reverse causality between BPHS and household consumption of rural migrant workers. Firstly, the fairer of BPHS, the more rights rural migrant workers enjoy in the destination, and the smaller the gap with urban residents, thus increasing household consumption of rural migrant workers. Secondly, the level of BPHS enjoyed by rural migrant workers not only depends on the local governments’ public service supply, but also the actual demand of rural migrant workers. In order to solve potential endogeneity problems caused by omitted unobservable variables and reverse causality, we used County−levelindex as instrumental variable (IV) for estimation. Specifically, we calculated this index by the following steps:

Firstly, calculate the proportion of rural migrant workers who obtain multidimensional BPHS in a county, which is used to represent the coverage breadth of multidimensional BPHS.


(3) $$\mathrm{Coverage}=\frac{\sum_{i=1}^{n}BPHS_{\text{\_}}\mathrm{equa}l_{i}}{n}$$

Secondly, calculate the weighted total number of rural migrant workers who obtain multidimensional BPHS, which is used to represent the depth of access to BPHS.


(4) $$\mathrm{Depth}=\frac{\sum_{i=1}^{n}BPHS_{\text{\_}}\mathrm{equa}l_{i}\times \mathrm{BPHS}\;\mathrm{inde}x_{i}}{\sum_{i=1}^{n}BPHS_{\text{\_}}\mathrm{equa}l_{i}}$$

Finally, calculate the equalization index of BPHS at county level.


(5) County−levelindex=Coverage×Depth

For the rationality of using county-level index as an instrumental variable, please refer to Supplementary material S2.

### 5.4 Mediation model

According to the hierarchical regression model (43), we assigned the regression models of BPHS to household consumption of rural migrant workers (see Equation 1), BPHS to health literacy or citizenization willingness, and BPHS and health literacy or citizenization willingness to household consumption of rural migrant workers. The last two equations were expressed as follows:


(6) $$\mathrm{Mediatio}n_{i}=\alpha_{2}+\beta_{2}\mathrm{BPHS}\;\mathrm{inde}x_{i}+\gamma_{2}\mathrm{Controls}+\theta_{p}+\varepsilon_{2i}$$

(7) $$\begin{array}{l} \mathrm{Consumptio}n_{i}=\alpha_{3}+\beta_{3}\mathrm{BPHS}\;\mathrm{inde}x_{i}+\mathrm{\delta Mediatio}n_{i}+\\ \gamma_{1}\mathrm{Controls}+\theta_{p}+\varepsilon_{3i} \end{array}$$

In Equations (6) and (7), $\mathrm{Mediatio}n_{i}$ indicates health literacy or citizenization willingness of the respondent i. $\beta_{2}$ and $\beta_{3}$ are the parameters to be estimated. $\varepsilon_{2i}$ and $\varepsilon_{3i}$ are the random disturbance terms.

The mediation effect test procedures include the following steps. Firstly, test the significance of $\beta_{1}$ in Equation (1). Continue to test if $\beta_{1}$ is significant, otherwise stop the test. Secondly, test the significance of $\beta_{2}$ in Equation (6) and δ in Equation (7). If both of them are significant, the mediation effect exists.

## 6 Results

### 6.1 Descriptive statistics

Table 3 reports the descriptive statistics of the above variables. In all rural migrant worker samples, the average of total household consumption is 43848.39 yuan, total household consumption without the conversion costs of accommodation and lodging is 41504.75 yuan, average person consumption is 15573.23 yuan, average labor consumption is 20673.93 yuan, consumption rate is 58.80%, and the average of total household consumption without housing expenditure is 34499.68 yuan. From the perspective of BPHS, the average BPHS index is 0.39 in the study sample, and the maximum and minimum values are 1 and 0, respectively.

For control variables, in terms of respondent characteristics, 59.00% of rural migrant workers are male, the average age is 36.62, education level is concentrated at the junior high school. Besides, 84.10% of respondents are married, only 3.60% of the samples are the members of the Communist Party of China, 54.90 and 38.00% of rural migrant workers are employed and self-employed, respectively. From the perspective of household characteristics, on average, family size is 3.19 and local size is 2.62. Older adult dependency ratio and child dependency ratio are 5.00 and 22.20%, respectively. As for household economic condition, the average of household income is 81619.55 yuan.

### 6.2 Baseline regression results

Table 4 reports the coefficients from regressions of BPHS on household consumption of rural migrant workers with OLS estimations. To be specific, column A does not contain any control variables, column B to column D gradually take control of respondent characteristics, household characteristics, and city fixed effects, respectively. All the coefficients on BPHSindex are significantly positive at the 1% level, which indicates that the results are robust. For the economic significance, according to column D, after controlling variables at respondent, household, and city fixed effects, the household consumption expenditure increases by 3.46% for every 1 unit increases in BPHS, verifying the hypothesis 1. Household of rural migrant workers consume more when they enjoy fairer treatments on BPHS in inflow cities. Therefore, enhancing BPHS is a key link and an important breakthrough to stimulate consumption and expand domestic demand.

**Table 4:** The impact of BPHS on household consumption of rural migrant workers: OLS estimations.

Independent variables	Consumption			
	A	B	C	D
BPHS index	0.125^***^ (0.0064)	0.052^***^ (0.0062)	0.032^***^ (0.0050)	0.034^***^ (0.0054)
Gender		0.005 (0.0034)	−0.005^*^ (0.0027)	−0.003 (0.0027)
Age		−0.007^***^ (0.0002)	−0.001^***^ (0.0002)	−0.001^***^ (0.0002)
Education		0.087^***^ (0.0019)	0.039^***^ (0.0015)	0.034^***^ (0.0016)
Married		0.447^***^ (0.0054)	0.047^***^ (0.0055)	0.043^***^ (0.0055)
Divorced or widowed		0.133^***^ (0.0126)	0.043^***^ (0.0104)	0.037^***^ (0.0103)
Party member		0.038^***^ (0.0091)	0.028^***^ (0.0073)	0.025^***^ (0.0072)
Employer		0.282^***^ (0.0074)	0.040^***^ (0.0060)	0.041^***^ (0.0061)
Self-employed		0.045^***^ (0.0036)	−0.002 (0.0029)	0.003 (0.0030)
Family size			0.017^***^ (0.0019)	0.022^***^ (0.0019)
Local size			0.040^***^ (0.0015)	0.036^***^ (0.0016)
Old share			0.015 (0.0362)	−0.017 (0.036)
Child share			0.088^***^ (0.0094)	0.092^***^ (0.0093)
Income			0.606^***^ (0.0028)	0.596^***^ (0.0030)
Constant	10.500^***^ (0.0031)	10.068^***^ (0.0112)	3.448^***^ (0.0312)	3.737^***^ (0.0534)
City FE	N	N	N	Y
Obs.	93,376	93,376	93,376	93,376
*Adj. R*^2^	0.004	0.119	0.434	0.454

Robust standard error is included in parentheses, and the significance level of 1, 5, and 10% are denoted by ***, **, and *, respectively.

Among the control variables, significantly negative coefficients are found on Age, indicating that older rural migrant workers spend less on consumption compared to younger people (5). The significantly positive coefficients on Education, Married, Divorcedorwidowed, Partymember, and Employer represent that consumption will increase when the respondent is an employer or a member of the Communist Party of China, not married, and have high level of education (4, 38). For household characteristics, lager family size or local size means higher consumption expenditure, which is similar to some previous studies (44). In terms of population burden ratio, there is a positive association between Childshare and consumption. Consistent with prior literature, positive effects are also existed in higher income households (41, 44).

### 6.3 Discussion on endogeneity

#### 6.3.1 The PSM estimation results

To further overcome the potential endogeneity problems in OLS estimations, we employed the propensity score matching (PSM) method. PSM is mainly applicable when the core explanatory variable is a binary variable. Based on this, we used BPHS_equal for further test.^6^ In this study, 1:2 nearest neighbor matching (NNM) and 0.05 caliper matching (CM) were adopted.

**Figure 5:**
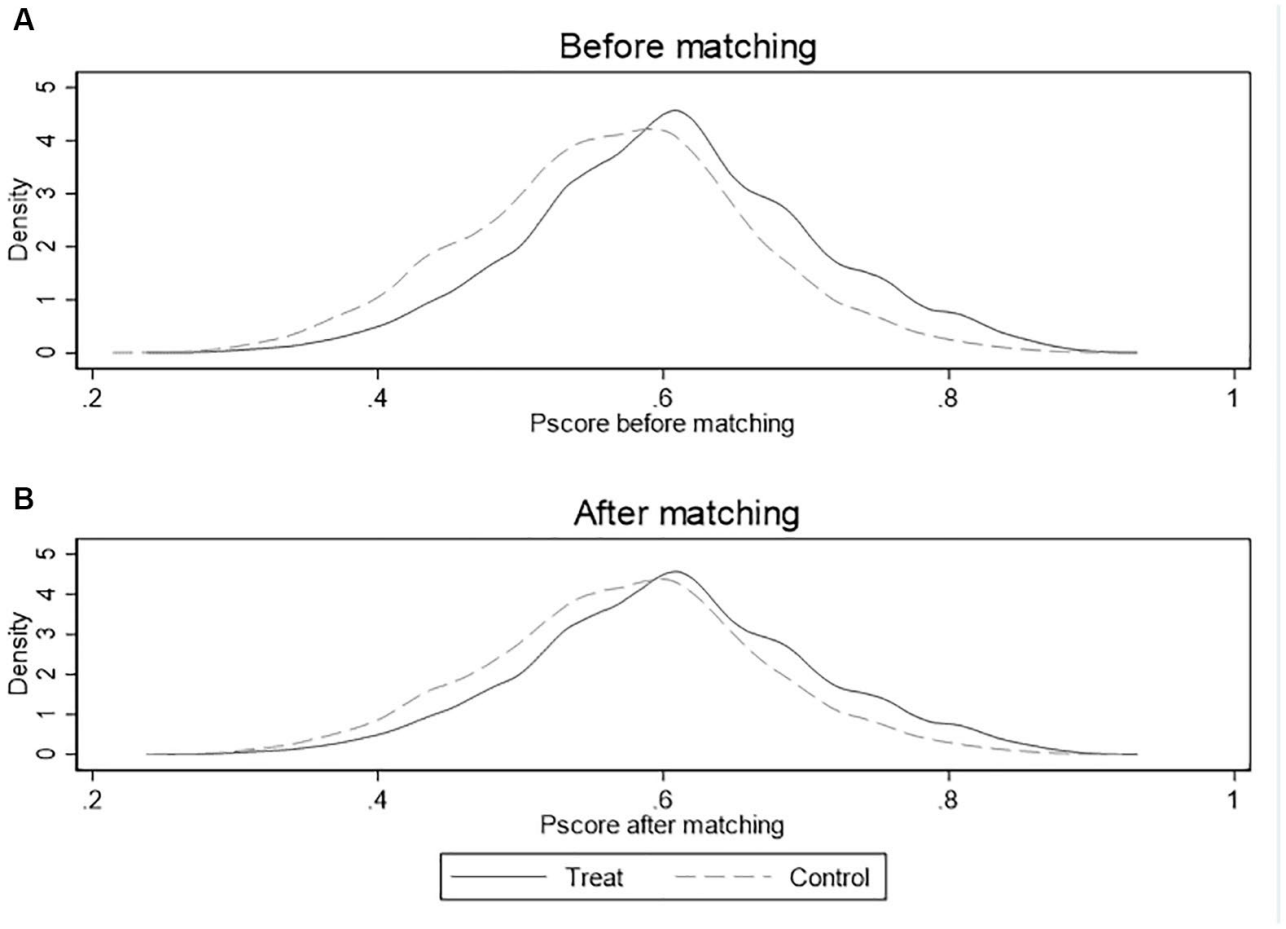
Common support assumption test for equalization situation density distribution of p-scores before and after matching in NNM. **(A)** Equalization situation before matching; **(B)** Equalization situation after matching.

**Figure 6:**
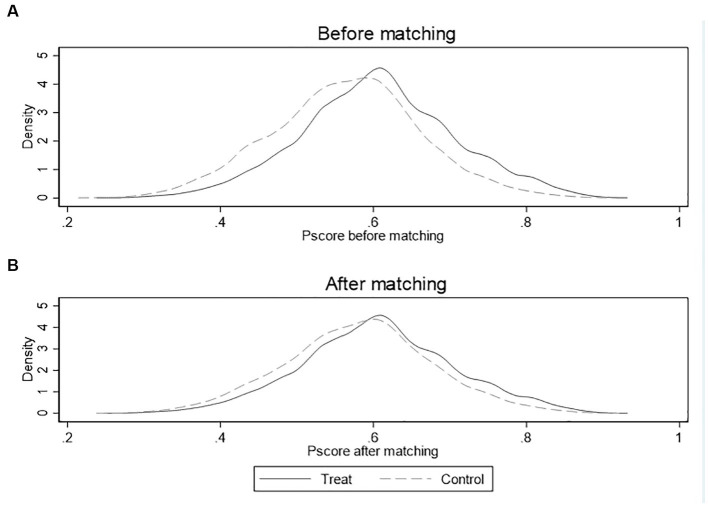
Common support assumption test for equalization situation density distribution of p-scores before and after matching in CM. **(A)** Equalization situation before matching; **(B)** Equalization situation after matching.

Figures 5, 6 show the kernel density of propensity scores before and after matching of NNM and CM, respectively. The horizontal axis represents the propensity score and the vertical axis represents the density value. We can find that the kernel density curves of the treatment group and control group before matching are significantly different, while the kernel density equation curves of two groups are much closer after matching. These changes indicate that the observable feature differences between the two groups are significantly reduced after matching, and the matching effects are good.

Table 5 reports the estimated results of PSM. The empirical results show that after correcting the self-selection bias of BPHS by using PSM method, BPHS has a significant positive effect on household consumption of rural migrant workers. To be specific, the estimated average treatment effect is 1.01 and 1.51% using the 1:2 nearest neighbor matching (NNM) and 0.05 caliper matching (CM) respectively. As a conclusion, the above results are consistent with the theoretical analysis, verifying the hypothesis 1.

**Table 5:** The impact of BPHS on household consumption of rural migrant workers: PSM estimations.

	NNM	CM
BPHS_equal	0.010^***^ (0.0033)	0.015^***^ (0.0038)
Constant	3.482^***^ (0.0462)	3.446^***^ (0.0526)
Control variables	Y	Y
City FE	Y	Y
Obs.	87,122	77,957
*Adj. R*^2^	0.435	0.435

Robust standard error is included in parentheses, and the significance level of 1, 5, and 10% are denoted by ***, **, and *, respectively.

#### 6.3.2 The IV estimation results

The instrument variable (IV) approach was also employed to address the potential endogeneity problems caused by omitted unobservable variables and reverse causality. As discussed in Supplementary material S2, we believed that County−levelindexwas an appropriate instrumental variable. The IV estimation results are shown in Table 6, where we include the same control variables and city fixed effects as in Table 4 column D. The *t* value of IV is significant at the 1% level and the first-stage F-statistics in the IV regression is 145.88, suggesting that this IV is a strong instrument, since it exceeds the conventional “rule of thumb” of 10 for an F-statistic.

**Table 6:** The impact of BPHS on household consumption of rural migrant workers: IV estimations.

	Consumption
BPHS index	0.044^***^ (0.0164)
Constant	3.735^***^ (0.0486)
Control variables	Y
City FE	Y
Obs.	93,160
*R*^2^	0.456
Wald *X*^2^	69527.190^***^
*F*-value of first stage	145.880^***^
*t* value of IV	116.810^***^

Robust standard error is included in parentheses, and the significance level of 1, 5, and 10% are denoted by ***, **, and *, respectively.

The results in Table 6 indicate that BPHS has a significantly positive impact on household consumption of rural migrant workers at the 1% level, which is consistent with the results of OLS estimations. While, it should be pointed out that the value of the IV coefficient is quite larger than that in OLS regressions, which is quite usual (45). The household consumption of rural migrant workers increases by 4.50% for every 1 unit increases in BPHS. Hence, we conclude that our main aforementioned conclusions are still confirmed with mitigating potential endogeneity of BPHS.

### 6.4 Robustness tests

We conducted several robustness checks.^7^ Firstly, we changed the measurements of consumption and used the logarithm of the total annual consumption expenditure of households excluding accommodation expenses (column A), the logarithm of *per capita* consumption (column B), the logarithm of consumption per worker (column C), the ratio of consumption to income (column D), as well as the logarithm of the total annual consumption expenditure without housing expenditure (column E). All the results are shown in Table 7, and the results remain robust.

**Table 7:** BPHS and different measurements of consumption: OLS estimations.

	A	B	C	D	E
	Consumption_exclude	Average person consumption	Average labor consumption	Consumption rate	Consumption without housing
BPHS index	0.010^*^ (0.0055)	0.034^***^ (0.0055)	0.032^***^ (0.0055)	0.012^***^ (0.0033)	0.049^***^ (0.0103)
Constant	3.263^***^ (0.0547)	4.196^***^ (0.0550)	4.223^***^ (0.0553)	3.078^***^ (0.0332)	3.601^***^ (0.1022)
Control variables	Y	Y	Y	Y	Y
City FE	Y	Y	Y	Y	Y
Obs.	93,376	93,376	93,376	93,376	93,376
Adj. *R*^2^	0.485	0.528	0.518	0.184	0.189

Robust standard error is included in parentheses, and the significance level of 1, 5, and 10% are denoted by ***, **, and *, respectively.

Secondly, we made several changes to the measurements of BPHS. Specifically, we used the BPHS_equal, which was calculated by the A-F double critical value method with the critical value of access to BPHS as 0.30 and 0.40, respectively, (column A and column B). We also replaced the original weight measured by the entropy method with an equal weight (column C) and reconstructed the index system by replacing certain indicators and their measurements (column D). Table 8 displays all the results, which remains robust despite these modifications.

**Table 8:** Different measurements of BPHS and consumption: OLS estimations.

	Consumption			
	A	B	C	D
BPHS index	0.012^***^ (0.0028)	0.011^***^ (0.0028)	0.022^***^ (0.0056)	0.031^***^ (0.0051)
Constant	3.737^***^ (0.0535)	3.738^***^ (0.0535)	3.732^***^ (0.0535)	3.740^***^ (0.0535)
Control variables	Y	Y	Y	Y
City FE	Y	Y	Y	Y
Obs.	93,376	93,376	93,376	93,376
*Adj. R*^2^	0.454	0.454	0.454	0.454

Robust standard error is included in parentheses, and the significance level of 1, 5, and 10% are denoted by ***, **, and *, respectively.

### 6.5 Mechanism analysis

#### 6.5.1 Improving health literacy

Health literacy refers to the ability of rural migrant workers to access, understand, and process health information and services to make decisions that benefit their own health. In other words, health behavior is a decision-making behavior based on the improvement of information acquisition ability and health cognition, which reflects health literacy to a certain extent. Based on the existing literature and data availability in the CMDS, respondents were asked to answer “when you were last sick (injured) or unwell, where did you first go for medical attention?,” the option “nowhere (no treatment)” was assigned to the value of 0 and others equaled to 1 (46). In order to make a more intuitive comparison of the differences in health literacy between rural migrant workers and urban residents, we further selected urban assimilation of health habits to discuss (47). To be specific, the questionnaire item “there is a big difference between my health habits and those of local citizens” was used to measure this indicator, options including fully agree, basically agree, disagree, and completely disagree, with a value of 1, 2, 3, and 4 in order. A higher score indicates higher health literacy.

Tables 9, 10 report the results of the mediation effect test of health literacy, which is measured by health behavior and health habit, respectively. We have already found the welfare effects of BPHS in column D of Table 4, with the magnitudes of 3.46%. As presented in column A of Tables 9, 10, BPHS affects health literacy positively and significantly at the 1% level. The coefficient on BPHSindex, Healthbehavior, and Healthhabitare all significantly positive at the 1% level in columns B, which is consistent with economic theoretical inferences and implies that the enhancement of BPHS does boost consumption by improving rural migrant workers’ health literacy. Besides, compared with the direct effect, the positive change after introducing health literacy is smaller. In conclusion, the hypothesis 2 is verified.

**Table 9:** Improving health literacy mechanism test results (health behavior).

	A	B
	Health behavior	Consumption
BPHS index	0.019^***^ (0.0064)	0.033^***^ (0.0054)
Health behavior	–	0.028^***^ (0.0028)
Constant	0.338^***^ (0.0633)	3.728^***^ (0.0535)
Control variables	Y	Y
City FE	Y	Y
Obs.	93,376	93,376
Adj. *R*^2^	0.090	0.455

(1) Robust standard error is included in parentheses, and the significance level of 1, 5, and 10% are denoted by ***, **, and *, respectively. (2) Health literacy is measured by health behavior.

**Table 10:** Improving health literacy mechanism test results (health habit).

	A	B
	Health habit	Consumption
BPHS index	0.189^***^ (0.0096)	0.031^***^ (0.0054)
Health habit	–	0.014^***^ (0.0018)
Constant	2.238^***^ (0.0958)	3.706^***^ (0.0536)
Control variables	Y	Y
City FE	Y	Y
Obs.	93,376	93,376
Adj. *R*^2^	0.076	0.455

(1) Robust standard error is included in parentheses, and the significance level of 1, 5, and 10% are denoted by ***, **, and *, respectively; (2) Health literacy is measured by health habit.

#### 6.5.2 Increasing citizenization willingness

Scholars commonly use psychological identity, long-term residence intention and hukou intention to measure citizenization willingness. In order to avoid errors and limitations in the measurement of a single index, this study selected indicators to measure the citizenization willingness of rural migrant workers from three aspects. (1) There were eight questions related to psychological identity in the CMDS.^8^ We divided agreement into four levels, ranging from completely disagree to completely agree on a scale of 1–4. The fifth through seventh sections were graded in reverse. Using equal weights, we then obtained the psychological identity score for each respondent, which ranged from 1 to 4; (2) In the CMDS 2017, statistics were made on the expected residence years of respondents in the local area. If rural migrant workers had the intention to live for more than 5 years, the value of Long−termresidenceintention was 1; otherwise, the value was 0; (3) According to the questions, “If you meet the requirements for local hukou, are you willing to move your hukou into the local government?,” if the answer was “yes,” the key variable Hukouintention equaled 1; otherwise, the value was 0. In terms of emotional level,Psychologicalidentity, Long−termresidenceintention, and Hukouintention show a progressive relationship.

Tables 11–13 summarize the outcomes of the mediation effect test of citizenization willingness, which is measured by psychological identity, long-term residence intention, and hukou intention, respectively. We have already verified that the enhancement of BPHS positively and significantly affects the household consumption of rural migrant workers. As presented in columns A of Tables 11–13, BPHS is positively and significantly associated with citizenization willingness which is measured by psychological identity, long-term residence intention, and hukou intention at the 1% level, with magnitudes of 0.257, 0.173, and 0.106, respectively. The coefficients on Psychologicalidentity, Long−termresidenceintention, and Hukouintention are all significantly positive at the 1% level in columns B. The results indicate that increasing citizenization willingness is one of the main channels through which the enhancement of BPHS boosts household consumption. Besides, after introducing citizenization willingness, the marginal effects of BPHSindex to Consumption are smaller than that in column D of Table 4. With reference to the mediation effect test procedures put forward by Baron and Kenny (43), we can conclude that the enhancement of BPHS can affect household consumption of rural migrant workers through the partial mediation effect of citizenization willingness, which verifies hypothesis 3.

**Table 11:** Increasing citizenization willingness mechanism test results (psychological identity).

	A	B
	Psychological identity	Consumption
BPHS index	0.257^***^ (0.0051)	0.029^***^ (0.0054)
Psychological identity	–	0.029^***^ (0.0035)
Constant	2.264^***^ (0.0504)	3.692^***^ (0.0541)
Control variables	Y	Y
City FE	Y	Y
Obs.	93,376	93,376
Adj. *R*^2^	0.159	0.455

(1) Robust standard error is included in parentheses, and the significance level of 1, 5, and 10% are denoted by ***, **, and *, respectively; (2) Citizenization willingness is measured by psychological identity.

**Table 12:** Increasing citizenization willingness mechanism test results (long-term residence intention).

	A	B
	Long-term residence intention	Consumption
BPHS index	0.173^***^ (0.0062)	0.023^***^ (0.0054)
Long-term residence intention	–	0.062^***^ (0.0028)
Constant	−1.138^***^ (0.0615)	3.808^***^ (0.0534)
Control variables	Y	Y
City FE	Y	Y
Obs.	93,376	93,376
Adj. *R*^2^	0.128	0.457

(1) Robust standard error is included in parentheses, and the significance level of 1, 5, and 10% are denoted by ***, **, and *, respectively; (2) Citizenization willingness is measured by long-term residence intention.

**Table 13:** Increasing citizenization willingness mechanism test result (hukou intention).

	A	B
	Hukou intention	Consumption
BPHS index	0.106^***^ (0.0061)	0.032^***^ (0.0054)
Hukou intention	–	0.018^***^ (0.0029)
Constant	0.180^***^ (0.0606)	3.734^***^ (0.0535)
Control variables	Y	Y
City FE	Y	Y
Obs.	93,376	93,376
Adj. *R*^2^	0.116	0.455

(1) Robust standard error is included in parentheses, and the significance level of 1, 5, and 10% are denoted by ***, **, and *, respectively; (2) Citizenization willingness is measured by hukou intention.

It would be interesting and clear to present a table with the hypotheses where it is established which are accepted. Table 14 summarizes the hypothesis test results and indicates that all the hypotheses we proposed above have been verified.

**Table 14:** Hypothesis test results.

Hypothesis	Results	Pass or not
Hypothesis 1	BPHS index↑ Consumption↑	Pass
Hypothesis 2	BPHS index↑ Health literacy↑ Consumption↑	Pass
Hypothesis 3	BPHS index↑ Citizenization willingness↑ Consumption↑	Pass

### 6.6 Heterogeneity analysis

#### 6.6.1 Heterogeneous effects by flow patterns

The theory of New Economic Geography emphasizes the important role of regional geographical location in the migration (48). Labor mobility is driven by rational economic decision-making, as individuals weigh the costs and benefits to maximize their own utility (49). Typically, workers in economically underdeveloped regions are more likely to migrate to areas with more job opportunities, higher incomes, and better public services, particularly across provincial or city boundaries (50). However, in some populous provinces with high numbers of rural migrant workers, those who only hold NRCMS in their hometowns face significant barriers to accessing medical services in other locations due to insurance reimbursement restrictions. In contrast, urban migrants are often able to more easily adapt and integrate into new cities, requiring less assistance from the host community. Fortunately, with the continuous advancement in BPHS, rural migrant workers are increasingly able to access the same medical insurance policies as urban residents in their destination cities, and the practice of settling medical insurance in different places has become more widespread across the country.

This study divided rural migrant workers into trans-provincial, intercity within the province and inter-country within the city according to their flow patterns, and explored the heterogeneity of the flow patterns of rural migrant workers’ household consumption caused by BPHS. Table 15 represents that the promotion effect of BPHS on household consumption of rural migrant workers mainly exists in trans-provincial and intercity within the province rural migrant workers, and the impact on trans-provincial rural migrant workers is higher than the overall average effect. However, the enhancement of BPHS does not promote household consumption among inter-country within the city migration. Taken together, these results indicate that the enhancement of BPHS has a higher marginal effect on improving the utility and welfare of the trans-provincial migration.

**Table 15:** Heterogeneous effects by flow patterns: OLS estimations.

	Trans-provincial	Intercity within the province	Intercountry within the city
BPHS index	0.047^***^ (0.0080)	0.023^**^ (0.0089)	0.006 (0.0125)
Constant	3.570^***^ (0.1166)	3.524^***^ (0.1450)	4.108^***^ (0.0924)
Control variables	Y	Y	Y
City FE	Y	Y	Y
Obs.	46,847	30,446	16,083
Adj. *R*^2^	0.434	0.492	0.477

Robust standard error is included in parentheses, and the significance level of 1, 5, and 10% are denoted by ***, **, and *, respectively.

#### 6.6.2 Heterogeneous effects by generations

Intergenerational difference is an important perspective in the study of rural migrant workers. Previous studies have divided rural migrant workers into two groups, the “old generation” and the “new generation,” based on whether they were born before or after 1980 (51). The “old generation” typically has lower levels of knowledge, skills, education, and weaker health conditions compared to the “new generation” (52). With sufficient provision of BPHS in inflow areas, rural migrant workers in the “old generation” can not only reduce their out-of-pocket health expenses but also alleviate their human capital deficiencies to a large extent (15), which ultimately leads to an increase in income and a decrease in precautionary savings, thereby improving their consumption level (14, 26). In contrast, the “new generation” faces more pressure to support their families and pay for mortgages. Although the enhancement of BPHS can improve their health literacy and increase their willingness to become citizens, their low income and high-pressure result in a lack of consumption power.

According to the conventional definition, we divided sample into old generation and new generation. The results of heterogeneous effects by generations are presented on Table 16. The coefficients on BPHSindex are significantly positive in both two groups, indicating that the enhancement of BPHS stimulates household consumption. In addition, the estimated average treatment effect is 4.81 and 1.31% in old generation and new generation respectively, reflecting the potentially positive role of BPHS for vulnerable groups with old generation.

**Table 16:** Heterogeneous effects by generations: OLS estimations.

	Old generation	New generation
BPHS index	0.047^***^ (0.0081)	0.013^**^ (0.0072)
Constant	3.737^***^ (0.0779)	3.787^***^ (0.0775)
Control variables	Y	Y
City FE	Y	Y
Obs.	43,736	49,640
Adj. *R*^2^	0.444	0.468

Robust standard error is included in parentheses, and the significance level of 1, 5, and 10% are denoted by ***, **, and *, respectively.

#### 6.6.3 Heterogeneous effects by income

There exists a positive correlation between income and consumption, which means that rural migrant workers’ households with higher income tend to have fewer budget constraints on consumption. These households are able to allocate more funds to areas such as culture, entertainment, and personal development (13). However, the primary goal of BPHS is to ensure the fundamental right of health, which is not directly linked to these consumption categories. Additionally, having more wealth provides greater certainty for the future, which can impact the welfare effect of BPHS (26). Therefore, while BPHS can have an impact on income and consumption, the marginal changes in income and consumption are not always apparent. As a result, it can be concluded that the enhancement of BPHS has a greater effect on improving the household consumption of lower-income levels compared to higher-income households.

Considering the large differences in the *per capita* disposable income of urban residents in different provinces, we compared the *per capita* disposable income of rural migrant workers with the income of urban residents in the destinations, and those higher than the income in the inflow cities were included into the higher-income group, the others were included into the lower-income group. Table 17 displays the results of heterogeneous effects by income. BPHS is positively and significantly associated with household consumption at the 1% level, with magnitudes of 3.67, and 2.53% in higher-income group and lower-income group, respectively. These findings suggest that the enhancement of BPHS plays a more significant role in improving the welfare of rural households with lower income, which reflects the inclusiveness of BPHS.

**Table 17:** Heterogeneous effects by income: OLS estimations.

	Lower income	Higher income
BPHS index	0.036^***^ (0.0062)	0.025^***^ (0.0107)
Constant	3.521^***^ (0.064)	4.313^***^ (0.1561)
Control variables	Y	Y
City FE	Y	Y
Obs.	66,861	26,515
Adj. *R*^2^	0.401	0.502

Robust standard error is included in parentheses, and the significance level of 1, 5, and 10% are denoted by ***, **, and *, respectively.

## 7 Discussion

With the ongoing process of urbanization, rural migrant workers have become an important part of China’s labor market (19). The growing consumer demand and influence wielded by these workers are reshaping the business landscape of Chinese cities, potentially facilitating the country’s transition to a consumption-driven economy (10). Particularly in the post-pandemic era, China and the global economy urgently require recovery. Therefore, how to transform rural migrant workers’ consumption potential into a consumption booster is crucial in promoting economic and social development and achieving common prosperity. Most studies have concentrated on the specific aspects of BPHS, and have proved that medical insurance significantly improves rural migrant workers’ household consumption. Enhancing BPHS is a necessary step toward deepening the reform of medical system, and promoting human well-being. While China has implemented a wide range of measures to advance the reform of BPHS, persistent challenges persist, including incomplete coverage, insufficient implementation, and uneven development (23, 24). With the increasing attention from policymaker, discussions about BPHS are becoming more widespread. However, these often overlook the circumstances surrounding rural migrant workers’ access to BPHS (18, 23, 44). As the “urban fringe population,” rural migrant workers are not only vulnerable to infectious diseases, but also more likely to become potential transmitters (53). Therefore, providing timely, comprehensive and effective BPHS to them is crucial, and may encourage them to increase household consumption with the improvement of health literacy and the increasement of citizenization willingness.

Using the data of CMDS 2017, this study firstly conducted an indicator system and applied the entropy method to measure rural migrant workers’ actual access to BPHS in inflow cities (39). Considering that household consumption was a continuous variable, we evaluated the impact of BPHS on the household consumption of rural migrant workers with OLS estimations, which was consistent with most of the existing literature (54, 55). The results show that after controlling respondent characteristics, household characteristics, and city fixed effects, the household consumption expenditure increases by 3.46% for every 1 unit increases in BPHS. Due to the availability of data and the presence of unique variables, we were compelled to utilize cross-sectional data, which can potentially introduce endogeneity problems. Therefore, referring to the methods of the existing literature (16, 56), we further employed the propensity score matching (PSM) method and the instrument variable (IV) approach to overcome the potential endogeneity problems in OLS estimations. Besides, we replaced the measurements of consumption and BPHS. The results are consistent with the baseline regression results. In the mechanism analysis, we used the hierarchical regression model, which is widely used in economic analysis, to verify the hypothesis 2 and 3 (43). The results show that improving health literacy and increasing citizenization willingness are two main channels through which the enhancement of BPHS boosts household consumption. Finally, we conducted grouping regression to examine heterogeneous effects (13). The positive effects of BPHS are more obvious in trans-provincial migration, old generation, and lower-income groups.

## 8 Conclusion

This study systematically explores the impact of BPHS on rural migrant workers’ household consumption by employing the OLS, PSM and IV methods. The results indicate that the enhancement of BPHS can boost household consumption of rural migrant workers, which expands the boundaries of consumption influencing factors and provides a new perspective for evaluating the welfare effects of policies. The mechanism analysis and heterogeneity analysis further clarify the internal logic and verify the inclusive function of BPHS, which supplements the field of the welfare of socially vulnerable groups, and provides targeted approaches to promote household consumption of rural migrant workers and achieve common prosperity.

The aforementioned findings have important policy implications. Firstly, the government should further enhance the equity of BPHS. By alleviating the burden of medical insurance and providing services such as free health check-ups and health education, the comprehensive access to services for rural migrant workers can be ensured. Consequently, this diminishes precautionary savings and medical expenditures, thereby fostering household consumption among rural migrant workers. Secondly, leveraging the welfare effects of BPHS is inclusive, and attention should be directed toward the needs of rural migrant workers who move across provinces, the older adult, and those with lower incomes. It is crucial to maximize the positive consumption externality of BPHS. Specifically, this can be achieved by improving awareness and utilization rates through effective publicity efforts. Additionally, actively guiding rural migrant workers to participate in community activities will help enhance their sense of belonging and identity in the city, thus improving their consumption in the inflow cities.

However, due to the data limitation, this study also has certain limitations. Firstly, this study exclusively focuses on the consumption of rural migrant workers and their cohabiting family members. However, economic ties with family members in their place of origin may introduce interference in the measurement of actual income and consumption. Secondly, the enhancement of BPHS may influence the lagged consumption of rural migrant workers. Additionally, apart from the promotional effect of BPHS, the consumption of rural migrant workers is also influenced by the high housing prices and living costs in the inflow cities. Lastly, considering the sample distribution and the focus on rural migrant workers in this study, the age range of the research subjects is restricted, potentially weakening the impact of digital literacy and specific diseases. Nevertheless, this study conducts a detailed discussion based on existing researches and undergoes a series of robustness tests to ensure the reliability of the conclusions, offering valuable insights into understanding this issue. In the presence of available data, further analysis can expand this topic using panel data to identify the time lagged effects of BPHS on consumption, taking inflow cities’ housing prices and living costs into consideration, and analyzing the heterogeneous impacts on different age groups after including rural migrant workers over the age of 65.

## Data availability statement

The data analyzed in this study is subject to the following licenses/restrictions: CMDS data needs to be simply requested on the official website. Requests to access these datasets should be directed to https://www.chinaldrk.org.cn/wjw/#/data/classify/population.

## Ethics statement

This study is a secondary analysis of the data from the CMDS. This survey was conducted by National Health Commission of China (https://www.chinaldrk.org.cn/wjw/#/home). All participants in the survey signed or marked (if illiterate) the informed consent forms.

## Author contributions

LP: Conceptualization, Data curation, Formal analysis, Investigation, Methodology, Project administration, Resources, Software, Supervision, Validation, Visualization, Writing – original draft, Writing – review & editing. GL: Conceptualization, Data curation, Project administration, Supervision, Validation, Writing – review & editing. HW: Conceptualization, Formal analysis, Resources, Validation, Writing – review & editing.

## Footnotes

^1^In this study, disease prevention and cure education includes occupational disease prevention, STD/AIDS control, tuberculosis control, and chronic disease prevention.

^2^In this study, health consciousness education includes reproductive health and contraception, smoking control, maternal and child health/eugenics, and public emergency self-rescue.

^12^Information source: https://health.gmw.cn/2022-07/15/content_35885951.htm

^12^Information source: http://www.gov.cn/guoqing/2022-03/23/content_5680879.htm

^5^Reasons for choosing this age range: (1) According to the Labor Law of the People’s Republic of China, it is prohibited for an employer to recruit minors under the age of 16. Therefore, we did not consider this group. (2) People over the age of 65 are defined as older adult according to international regulations. From the data distribution in the available reports, the number of rural migrant workers over 65 years old is small, which is consistent with reality. In general, if the sample distribution is particularly dispersed, including people over the age of 65 in the study may adversely affect the regression results. Therefore, rural migrant workers over the age of 65 were not considered in this study.

^6^In this analysis, we set the critical value of access to BPHS as 0.30 to construct the variable BPHS_equal.

^7^Due to length limitations, detailed interpretation of the robustness test results can be found in Supplementary material 3.

^8^Eight questions are presented as follows. (1) I like the city/place where I live now; (2) I pay attention to the changes in the city/place where I live now; (3) I would love to blend in with the locals and be one of them; (4) I think the locals are willing to accept me as one of them; (5) I feel that locals look down on outsiders; (6) It’s important for me to follow the customs of my hometown; (7) My hygiene habits are quite different from those of the local people; (8) I feel like I’m already a local person.
