# Supplementary material for: Does improving basic public health services promote household consumption of rural migrant workers? Evidence from China
Source: Front Public Health. 2024 Jan 8;11:1308297. doi: 10.3389/fpubh.2023.1308297 (PMC10800378; doi:10.3389/fpubh.2023.1308297)
Supplement: Supplementary file 1 [file Table_1.DOCX]

Supplementary Material

1. **Institutional background of BPHS reform in China**

China has attached great importance to basic public services, especially in the field of BPHS. The development processes of BPHS can be roughly divided into three periods, including initial period, exploration and development period, and stabilization and standardization period.

**Initial period.** In the early 1950s, China’s primary health service system was initiated. In the following decades, China has lunched market-oriented reforms on medical and health service system and a series of insurance schemes, such as the Urban Employee Basic Medical Insurance (UEBMI), the New Rural Cooperative Medical Scheme (NRCMS), and the Urban Resident Basic Medical Insurance (URBMI).

**Exploration and development period.** In 2009, in order to deepen the reform of medical system, six major public health service projects were launched. Besides, the National Basic Public Health Service Program (NBPHS) was issued by the Ministry of Health (MOH), nine services in three categories were provided free of charge to urban and rural residents, including establishment of residents’ health files, health education, child health care, maternal health care, elderly health care, vaccination, prevention and control of infectious diseases, chronic disease management, and severe mental illness management. According to the actual demand of residents, the feasibility and effects of implementation, China has continuously enriched NBPHS while considering the maximum support capacity of the government finance. Now, NBPHS include fourteen items, added tuberculosis management, TCM health management, free provide for contraceptive, and promotion action of health literacy.

**Stabilization and standardization period.** In 2012, The State Council issued the document “12th Five-Year Plan for the Development of health Services”, which aimed to ensure that by 2015 all residents would have access to basic medical security and BPHS. In 2013, the “National Health and Family Planning Commission of China” (NHFPC) was established by restructuring the MOH and the Population and Family Planning Commission, and then the first and novel internal migrant-targeted public health policy was carried out in November. In the “Equalization Program of Basic Public Health and Family Planning Services for Migrants” (EHFPSM), forty cities among twenty-seven provinces were assigned as the pilots, seven major services were provided free to migrants. In the period of the 14th Five-Year Plan, increasing the degree of approximation of basic public services and increasing the satisfaction of the general public with the quality of a good life are the most fundamental conditions for common prosperity. And the report to the 20th National Congress of the Communist Party of China proposed to improve the system of basic public services, improve the level of public services, make them more balanced and accessible, and steadily promote common prosperity.

1. **Rationality of County-level index**

$County-level equalization index$

$$\begin{aligned} Coverage=\frac{\sum_{i=1}^{n} {BPHS_{e}qual}_{i}}{n}\#\left( 1 \right) \end{aligned}$$

$$\begin{aligned} Depth=\frac{\sum_{i=1}^{n} {BPHS_{e}qual}_{i}\times{BPHS index}_{i}}{\sum_{i=1}^{n} {BPHS_{e}qual}_{i}}\#\left( 2 \right) \end{aligned}$$

$$\begin{aligned} County-levelequalizationindex=Coverage\times Depth\#\left( 3 \right) \end{aligned}$$

There are two reasons to choose this instrumental variable. Firstly, county-level index is highly correlated with individual BPHS acquisition. Studies have shown that under China’s current decentralization system, county-level governments bear the main responsibility for providing public services, especially in the areas of basic education, health care and social security. The enhancement of BPHS in county can provide different preferences of public service combinations for the flow and allocation of factors within and between regions, and then increase residents’ satisfaction. Secondly, county-level index is basically determined by policy arrangement and allocation of financial funds, and this variable is relatively macro, which has a weak impact on the consumption of micro individuals. In other words, this variable satisfies exclusivity.

As a conclusion, the two requirements, correlation and exogeneity, for a valid instrument are likely to be satisfied. $County-level equalization index$ is an appropriate instrumental variable.

1. **Robustness test results**

We conducted several robustness checks. First, we took the logarithmic of household’s total annual consumption expenditure without the conversion cost of accommodation and lodging as dependent variable. The results in table 7 column A show that the coefficient on $BPHS index$ is significantly positive, indicating that whether the converted amount of food and housing covered by employment units are included or not, our findings remain unchanged.

Second, we used the logarithmic of average person consumption, and the logarithmic of average labor consumption to replace the logarithmic of total consumption. The results in column B and C of table 7 indicate that average person consumption and average labor consumption increase by 3.46% and 3.25% for every 1 unit increases in BPHS respectively. In other words, regardless of whether family size or labor force size is considered, the main conclusions above are robust.

Third, we further considered the ratio of household consumption to household income. The empirical results are shown in column D of table 7. The coefficient on $BPHS index$ is still significantly positive at the 1% level. This result confirms the important role of BPHS enhancement in reducing precautionary savings and promoting consumption once again.

Fourth, we took the logarithmic of household’s total annual consumption expenditure without housing expenditure as dependent variable. The results in table 7 column E show that the coefficient on $BPHS index$ is significantly positive, indicating that the enhancement of BPHS promotes household consumption of rural migrant workers even after excluding the impact of housing costs.

**Table 7** BPHS and different measurements of consumption: OLS estimations

|  | **A** | **B** | **C** | **D** | **E** |
| --- | --- | --- | --- | --- | --- |
|  | **Consumption_exclude** | **Average person consumption** | **Average labor consumption** | **Consumption rate** | **Consumption without housing** |
| BPHS index | 0.010^*^  (0.0055) | 0.034^***^  (0.0055) | 0.032^***^  (0.0055) | 0.012^***^  (0.0033) | 0.049^***^  (0.0103) |
| Constant | 3.263^***^  (0.0547) | 4.196^***^  (0.0550) | 4.223^***^  (0.0553) | 3.078^***^  (0.0332) | 3.601^***^  (0.1022) |
| Control variables | Y | Y | Y | Y | Y |
| City FE | Y | Y | Y | Y | Y |
| Obs. | 93376 | 93376 | 93376 | 93376 | 93376 |
| *Adj. R^2^* | 0.485 | 0.528 | 0.518 | 0.184 | 0.189 |

Notes: robust standard error is included in parentheses, and the significance level of 1%, 5%, and 10% are denoted by ***, ** and *, respectively.

Fifth, we used the independent variable $BPHS\_equal$ calculated by A-F double critical value method with the critical value of access to BPHS as 0.30 and 0.40 respectively. The results in column A and column B of table 8 are qualitatively similar to the main estimates, although somewhat smaller in magnitude.

Sixth, we employed equal weight to replace original weight measured by the entropy method. In column C of table 8, we find that the coefficient on $BPHS index$ is significantly positive at the 1% level. To be specific, the estimated average treatment effect is 2.22%. By replacing the calculation method of weight, we can still draw the conclusion that the enhancement of BPHS promotes household consumption of migrant workers.

Lastly, in order to reduce the unreliability of the results caused by the subjectivity of index selection, this study reconstructed the index system by replacing some indicators and their measurements. To be specific, there were three changes as follows: (1) considering that public health file was not yet fully available, migrant workers who have not built health file but have heard of it was also regarded as obtaining this right; (2) the way of health education will greatly affect the actual effect. This study argued that health knowledge lectures, public health consultation activities, and individualized face-to-face consultation presented more obvious effects. Therefore, we adopted a new indicator $Education method$ to replace three kinds of health education, and the value of the above health education methods equaled 1; otherwise, the value was 0; (3) if migrant workers participated in any medical insurance at the inflow cities, the key indicator $Medical insurance$ equaled 1; otherwise, the value was 0. As shown in column D of table 8, the household consumption expenditure increases by 3.15% for every 1 unit increases in BPHS. In other words, the results in this study are robust.

**Table 8** Different measurements of BPHS and consumption: OLS estimations

|  | **Consumption** | | | |
| --- | --- | --- | --- | --- |
|  | **A** | **B** | **C** | **D** |
| BPHS index | 0.012^***^  (0.0028) | 0.011^***^  (0.0028) | 0.022^***^  (0.0056) | 0.031^***^  (0.0051) |
| Constant | 3.737^***^  (0.0535) | 3.738^***^  (0.0535) | 3.732^***^  (0.0535) | 3.740^***^  (0.0535) |
| Control variables | Y | Y | Y | Y |
| City FE | Y | Y | Y | Y |
| Obs. | 93376 | 93376 | 93376 | 93376 |
| *Adj. R^2^* | 0.454 | 0.454 | 0.454 | 0.454 |

Notes: robust standard error is included in parentheses, and the significance level of 1%, 5%, and 10% are denoted by ***, ** and *, respectively.
